# Engineering the next generation of allogeneic CAR cells: iPSCs as a scalable and editable platform

**DOI:** 10.1016/j.stemcr.2025.102515

**Published:** 2025-06-05

**Authors:** Ying Fang, Yuning Chen, Yan-Ruide Li

**Affiliations:** 1 Department of Microbiology, Immunology & Molecular Genetics, University of California, Los Angeles, Los Angeles, CA 90095, USA; 2 Department of Bioengineering, University of California, Los Angeles, Los Angeles, CA 90095, USA

**Keywords:** induced pluripotent stem cell, iPSC, chimeric antigen receptor, CAR, cancer, immunotherapy, allogeneic cell therapy, clinical trials, CRISPR

## Abstract

Over the past five years, allogeneic off-the-shelf CAR-engineered cell therapies have advanced rapidly. By bypassing the individualized manufacturing, high cost, and eligibility constraints of autologous products, allogeneic platforms, especially those derived from induced pluripotent stem cells (iPSCs), promise broader, faster access for cancer patients. This perspective reviews recent preclinical and clinical milestones, outlining genetic designs, scalable production workflows, and early-phase trial outcomes. We assess safety profiles, antitumor activity, and *in vivo* persistence, spotlighting innovations like T cell receptor alpha constant (*TRAC*) knockout, human leukocyte antigen (HLA) camouflage, and interleukin (IL)-15 armoring. Finally, we identify emerging trends and challenges that will shape the future development of allogeneic iPSC-derived CAR therapies.

## Teaser

Li and colleagues underscore the potential of iPSCs as a foundational platform for allogeneic cell therapies. They provide an overview of recent clinical advancements in iPSC-derived CAR-based therapies for cancer, summarizing ongoing clinical trials and preclinical developments. They also discuss key aspects of genetic engineering strategies, clinical methodologies, and reported efficacy and safety outcomes associated with these emerging therapeutic modalities.

## Introduction

Chimeric antigen receptor-engineered T (CAR-T) cell therapy has revolutionized cancer treatment, particularly for hematologic malignancies. To date, seven CAR-T cell therapies have received Food and Drug Administration (FDA) approval, targeting conditions such as B cell precursor acute lymphoblastic leukemia (B-ALL), large B cell lymphoma, follicular lymphoma, mantle cell lymphoma, and multiple myeloma (MM). Notably, all these FDA-approved therapies utilize autologous T cells. Despite their clinical success, autologous CAR-T therapies face significant challenges. The manufacturing process is labor intensive and time consuming, often leading to delays that may cause patients to miss critical therapeutic windows. Additionally, many patients have compromised immune systems due to prior chemotherapy or the underlying disease, resulting in insufficiently robust T cells for effective gene engineering and expansion. Furthermore, the cost of autologous CAR-T therapies is prohibitively high, with prices ranging from around $300k to around $500k per dose. These limitations underscore the urgent need for readily available, cost-effective, off-the-shelf cell products. Allogeneic CAR-T therapies, derived from healthy donors, offer a promising alternative. In this perspective, we examine recent clinical and preclinical studies on allogeneic CAR-T cell therapies, discuss their current limitations, and explore how induced pluripotent stem cells (iPSCs) may provide innovative solutions, potentially guiding the future direction of this field.

## Sources for allogeneic car cell product

Allogeneic CAR-T cell therapies can be derived from four primary cellular sources, each with distinct advantages and limitations. The most common source is third-party donor peripheral blood T cells, obtained from healthy adult donors through leukapheresis (Li et al., 2022). This approach provides a readily available supply of high-quality, functionally mature T cells, which can be expanded and genetically modified for therapeutic use. To mitigate the risk of graft-versus-host disease (GvHD), endogenous T cell receptors (TCRs) are typically disrupted through gene editing (Lonez and Breman, 2024). This strategy has been widely adopted in clinical allogeneic CAR-T cell therapies. For example, in the UCART19 clinical trial, the T cell receptor alpha constant (*TRAC)* gene in these therapeutic CAR-T cells was disrupted using mRNA encoding transcription activator-like effector nucleases (TALENs) to reduce GvHD (Benjamin et al., 2022). In ALLO-715 CAR-T therapy for MM, the TCR alpha chain was disrupted using the same method (Sommer et al., 2018). Umbilical cord blood (UCB) represents another potential source. UCB-derived T cells are advantageous due to their high proportion of naive T cells and reduced immunogenicity, which lowers the risk of GvHD (Liu et al., 2022). Additionally, UCB units are readily available from cord blood banks, offering a convenient supply of cells. However, each unit contains only a limited number of T cells, often requiring cells from multiple donors to generate sufficient numbers for therapy (Yu et al., 2023). Furthermore, UCB-derived cells engraft and reconstitute the immune system more slowly than peripheral blood T cells, which may affect their therapeutic efficacy. Hematopoietic stem and progenitor cells (HSPCs) provide a renewable source of T cells with defined properties through directed differentiation. This approach allows for the generation of CAR-T cells with specific phenotypic and functional characteristics. However, differentiation protocols are complex and time consuming, and ensuring the safety and efficacy of the final CAR-T cell product remains a significant challenge (Jing et al., 2025; Netsrithong et al., 2024).

A rapidly emerging alternative is the use of iPSCs as a source for CAR-T cell therapy. iPSCs, generated through the reprogramming of somatic cells, possess the ability to differentiate into any cell type, including T cells (Iriguchi et al., 2021; Netsrithong et al., 2024). This approach offers the potential for a standardized, renewable, and scalable source of CAR-T cells with controlled genetic modifications. iPSCs possess the capacity for indefinite self-renewal, allowing for the generation of large quantities of T cells from a single, well-characterized clone, which contrasts with HSPCs that have limited proliferative potential and can exhibit variability between donors (Laurenti and Göttgens, 2018; Netsrithong et al., 2024). iPSCs are also highly amenable to precise genetic modifications and can be engineered to express an exogenous TCR. During T cell differentiation, the transgenic TCR generates a CD3 signal, which leads to allelic exclusion and exclusive expression of the transgene, thereby providing a safer profile (Netsrithong and Wattanapanitch, 2021). Additionally, iPSCs can be reprogrammed from healthy donor T cells, retaining the epigenetic memory of their original T cell identity. This characteristic facilitates more efficient and directed redifferentiation into functional T cells compared to iPSCs derived from other somatic cell types, such as fibroblasts (Iriguchi et al., 2021; Loh et al., 2010). Moreover, iPSCs reprogrammed from T cells maintain pre-rearranged TCRα and TCRβ loci, which enables the regeneration of monoclonal, antigen-specific T cells, provided that the original TCR is preserved (Minagawa et al., 2018). However, challenges such as efficient differentiation into functional T cells, tumorigenicity risks, and optimizing manufacturing protocols remain key areas of ongoing research.

## Current clinical trials with allogeneic third-party donor CAR cell products

Currently, the clinical trials have explored the use of third-party donor T cells, natural killer (NK) cells, and natural killer T (NKT) cells for allogeneic cell therapy. Additionally, lymphocytes differentiated from genetically engineered stem cells, such as HSPCs, are being investigated as alternative sources. In these therapies, CARs are engineered to target specific antigens. A variety of CAR constructs have been evaluated in allogeneic settings, targeting antigens such as CD19, CD22, CD7, B cell maturation antigen (BCMA), and CD70 (Lekakis et al., 2021; Locke et al., 2025; McGuirk et al., 2022; Ortí et al., 2025; Zhou et al., 2025). Dual-targeting strategies, including CD19/CD20 and CD19/CD22 CAR-T cells, have been employed to enhance therapeutic efficacy and mitigate antigen escape (Hu et al., 2021; Phely et al., 2024). These therapies are being investigated in hematologic malignancies, including B-ALL, relapsed/refractory large B cell lymphoma, MM, and acute myeloid leukemia (AML). Expanding beyond hematologic applications, allogeneic CAR-based therapies have also been evaluated in solid tumors such as advanced clear cell renal cell carcinoma, as well as refractory immune-mediated conditions like necrotizing myopathy and systemic sclerosis (Li et al., 2024; Pal et al., 2024). Notably, innovative CAR designs have been developed to optimize CAR-T cell fate and functionality. For example, CARs encoding a single immunoreceptor tyrosine-based activation motif (ITAM) can direct T cells toward distinct fates by balancing effector and memory programs, thereby enabling the generation of CAR constructs with enhanced therapeutic profiles and improving both the efficacy and persistence of CAR-T cells (Feucht et al., 2019; Nishimura et al., 2013; Ueda et al., 2023).

To enhance the safety and efficacy of these therapies, gene-editing strategies are employed. Techniques such as TALENs or CRISPR-Cas9 are used to knock out TCRs, thereby preventing GvHD. Knockout (KO) of human leukocyte antigen (HLA) class I and II molecules reduces host immune recognition, thereby enhancing persistence (Chen et al., 2023a). CAR constructs are frequently integrated into the *TRAC* locus to ensure stable expression, and safety switches such as RQR8 or inducible Cas9 (iCas9) provide a means for controlled cell elimination if needed (Benjamin et al., 2022). However, emerging evidence indicates that disruption of the endogenous TCR may impair CAR-T cell persistence and antitumor activity, potentially due to the absence of tonic TCR signaling and associated compensatory mechanisms (Li et al., 2025b; Stenger et al., 2020). These findings underscore the need for careful consideration when engineering TCR-deficient CAR-T cells, particularly in the context of long-term efficacy. Additional modifications, such as CD52 KO to confer resistance to alemtuzumab and overexpression of NK-inhibitory ligands (e.g., HLA-E and CD47), further enhance immune evasion and therapeutic durability (Benjamin et al., 2020; Lekakis et al., 2021; Neelapu et al., 2021).

## Limitations of allogeneic CAR-based therapy

### Batch-to-batch variability

A key limitation of allogeneic CAR therapies is batch-to-batch variation due to differences in donor genetics, immune status, and overall cell quality. Peripheral blood-derived T cells from different donors exhibit significant heterogeneity in phenotype, cytokine production, and expansion potential, which can impact the consistency of the final therapeutic product (Ghosh et al., 2017). For instance, donor age and health status influence the proliferative capacity of T cells, with younger donors typically providing more robust and persistent T cell products (Brudno and Kochenderfer, 2019). Genetic variability in baseline immune activation further contributes to inconsistencies in the starting material, posing challenges in standardizing allogeneic CAR-T cell manufacturing. This inherent variability may affect product uniformity, ultimately impacting therapeutic efficacy and safety.

### Safety and immunogenicity concerns

Allogeneic CAR-T cells require additional genetic modifications to mitigate GvHD and allorejection by the host immune system (Li et al., 2024). A widely adopted strategy involves disrupting the endogenous TCR using CRISPR-Cas9 or TALEN gene editing, effectively preventing GvHD by eliminating the ability of donor-derived T cells to recognize host antigens (Chen et al., 2023a). To further reduce alloreactivity, additional modifications target the deletion of HLA class I and class II molecules, thereby evading host T cell-mediated rejection (Chen et al., 2023a). Despite these modifications, allogeneic CAR-T cells remain susceptible to clearance by innate immune effectors, particularly NK cells (Chen et al., 2023a). Strategies such as the overexpression of inhibitory ligands, including HLA-E and CD47, have been explored to shield CAR-T cells from NK cell-mediated cytotoxicity (Chen et al., 2023a). Additionally, the KO of immune checkpoint molecules, such as PD-1, has been investigated as a means to enhance CAR-T cell persistence and antitumor function by reducing T cell exhaustion (Ren et al., 2017). However, extensive genetic modifications introduce potential risks, including off-target effects and genomic instability, which raise safety concerns that must be addressed in clinical translation. CRISPR-Cas9-mediated gene editing can inadvertently introduce mutations at unintended genomic sites due to the similarity of the genomes, leading to potential disruptions in gene function or activation of oncogenes (Kang et al., 2020; Lei et al., 2024). To mitigate these risks, strategies such as optimizing guide RNA design and selecting high-fidelity engineered nucleases are under investigation (Kim et al., 2019; Madison et al., 2022). Addressing these safety concerns is critical for the successful clinical translation of allogeneic CAR-T cell therapies.

### Challenges in multiplex gene editing

While multiplex gene editing is a promising strategy for enhancing the functionality and persistence of allogeneic CAR-T cells, achieving efficient and accurate multiplex gene editing is technically demanding. The genomic instability is caused by multiplex gene editing (Webber et al., 2019). In allogeneic cell therapy, T cells often undergo additional gene editing alongside CAR integration for improved efficacy and safety. However, approaches such as CRISPR gene editing can induce DNA double-strand breaks, which may lead to chromosomal aberrations including large segment deletions, inversions, and translocations (Lei et al., 2024; Liu et al., 2017; Ren et al., 2017). For example, CRISPR ribonucleoprotein editing of the *TRAC* locus in human primary T cells has shown that approximately 5%–20% of modified T cells exhibit large segmental or whole deletions of chromosome 14 (Tsuchida et al., 2023). To mitigate these risks, alternative gene-editing strategies that avoid double-strand breaks, such as base editing and prime editing, are under investigation for their potential to introduce precise genetic modifications with reduced genomic instability (Pomeroy et al., 2023; Webber et al., 2019). Additionally, optimizing the timing of T cell activation relative to gene editing has shown promise. Since MDM2 expression following T cell activation suppresses p53’s repair capacity, editing non-activated T cells may reduce the incidence of large deletions and chromosomal translocations compared to editing activated T cells (Tsuchida et al., 2023). Minimizing these risks in multiplex gene editing is essential for ensuring the safety and efficacy of next-generation allogeneic CAR-T cell products.

### Donor consent and ethical concerns

The use of allogeneic cell sources introduces ethical and regulatory complexities, particularly concerning donor consent and cell banking for future therapeutic applications. UCB, HSPCs, and iPSCs represent emerging sources for allogeneic CAR therapies, yet ensuring proper informed consent from donors remains a critical requirement. UCB is often collected at birth, with parental consent obtained under circumstances that may not allow for fully informed decision-making (Nagamura-Inoue and Nagamura, 2023). Additionally, the use of banked UCB for commercial therapies raises questions about donor rights and the ownership of biological materials. Similarly, the collection of HSPCs from adult donors involves ethical considerations regarding donor autonomy, potential risks associated with mobilization procedures, and the adequacy of informed consent. The expansion of allogeneic CAR therapies also intensifies concerns about the limited availability of matched donors, which may exacerbate disparities in access to advanced cellular therapies.

## iPSC renders possible solutions

**Figure 1:**
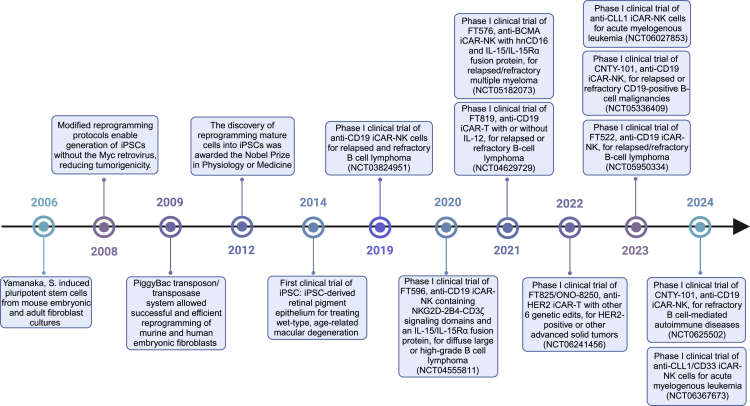
Timeline of iPSC development including iPSC-derived CAR-engineered cell therapy clinical trials

Advancements in iPSC research have established a robust platform for allogeneic CAR-engineered cell therapies, addressing major limitations of traditional cell therapies (Figure 1).

### Unlimited source

A major challenge in adoptive cell therapy is the dependence on patient-derived or donor-sourced cells, which suffer from inter-donor variability, batch-to-batch inconsistencies, and limited expansion potential (Furukawa et al., 2022; Lindenbergh and van der Stegen, 2025; Nicholson et al., 2022). iPSCs circumvent these limitations by providing an essentially limitless source of cells, capable of differentiation into various immune cell types (Lindenbergh and van der Stegen, 2025; Sebastiano et al., 2014). The development of HLA-matched or engineered universal donor iPSC banks further enhances their clinical applicability by reducing immune rejection and allowing standardized cell therapy production (Goldenson et al., 2022; Harrison et al., 2019; Madrid et al., 2024; Shin et al., 2025). Moreover, rigorous quality control measures, including single-cell RNA sequencing and high-throughput imaging techniques, ensure the reproducibility and consistency of iPSC-derived products (Madrid et al., 2024; Mehta et al., 2022b). Potency assays are also required to determine whether the product release has the functional capacity to achieve the desired clinical effect (Madrid et al., 2024). This standardization is critical for generating homogeneous cell therapy products that meet regulatory and clinical safety benchmarks.

### Differentiation to low GvHD and immunogenicity cell types

Another essential criterion for determining the therapeutic cell product is the risk of GvHD and allorejection (Benjamin et al., 2020; Li et al., 2024). iPSC-derived conventional T cells possess a diverse TCR repertoire, enabling recognition of host major histocompatibility complex (MHC) molecules, which can potentially lead to GvHD (Jing et al., 2022; Montel-Hagen et al., 2019). In contrast, iPSCs can be directed to differentiate into immune cell subsets with inherently improved safety profiles, such as unconventional T cells (e.g., NKT and γδ T cells) and NK cells, which are not restricted by MHC-mediated antigen recognition, thereby reducing the risk of alloreactivity and supporting the development of off-the-shelf allogeneic immunotherapies (Chen et al., 2023b; Fang et al., 2023; Li et al., 2025a, 2025c; Shin et al., 2025). Additionally, genetic engineering approaches have facilitated the generation of hypoimmunogenic iPSC-derived cells by modifying key immune recognition pathways. For example, knocking out *B2M* disrupts the MHC class I molecule presentation, reducing cytotoxic T cell-mediated rejection, while engineering strategies maintaining HLA-C at the same time help mitigate NK cell-mediated allorejection (Li et al., 2021; Nicholson et al., 2022).

### Avoid ethical concerns

Unlike embryonic stem cells (ESCs), which raise ethical concerns due to their derivation from human embryos, iPSCs are reprogrammed from somatic cells, avoiding ethical dilemmas (Yamanaka, 2020). This attribute allows broader acceptance and regulatory approval for iPSC-based cell therapies. Additionally, the ability to generate both autologous or allogeneic iPSC lines further supports their ethical and clinical feasibility (Mehta et al., 2022a; Wang et al., 2024).

## Current clinical trials with iPSC-derived CAR cell products

**Figure 2:**
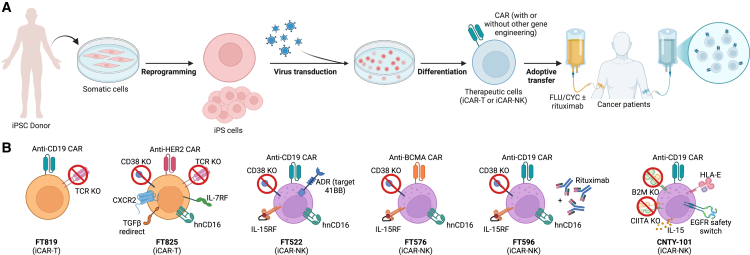
iPSC-derived CAR cell products in clinical development (A) Schematic representation of the generation of iPSC-derived CAR-engineered immune cells and their application in clinical trial designs. (B) Current therapeutic strategies for iPSC-derived CAR-T and CAR-NK cell therapies, highlighting key design elements.

Current iPSC-derived CAR-engineered (iCAR) cell therapies primarily include two major platforms: iCAR-T cells and iCAR-NK cells (Figure 2). These approaches utilize diverse CAR constructs targeting CD19 and HER2 for iCAR-T cells, while iCAR-NK cells incorporate CARs recognizing CD19, BCMA, CCL1, and CD33. The therapeutic landscape for iCAR therapies extends beyond hematologic malignancies, including B cell malignancies and AML to autoimmune diseases such as systemic lupus erythematosus and even advanced solid tumors.

The emerging iPSC-derived CAR portfolio shows a clear correspondence between each product’s molecular “build” and the clinical phenotype observed in first-in-human trials. FT819, in which a CD19 1XX CAR is precisely knocked into the *TRAC* locus while ablating the endogenous TCR to avert GvHD, has produced early antitumor activity in B cell malignancies and sustained B cell depletion in systemic lupus erythematosus (Mehta et al., 2022a). By contrast, the iPSC-derived, off-the-shelf NK cell product FT522 features CD38 knockout, a non-cleavable high-affinity CD16 receptor, and an interleukin (IL)-15/IL-15Rα fusion, in addition to a CD19 CAR for the treatment of B cell lymphoma (Bachanova et al., 2024). The iPSC-derived CAR-NK cell FT596 with the same gene editions as FT522 demonstrated long-term responses across multiple lymphoma subtypes, including patients previously treated with CAR-T therapy, suggesting a recommended phase 2 dose of 1.8 × 10^9^ cells (Bachanova et al., 2021; Ghobadi et al., 2025). FT516 carrying the hnCD16/IL-15 module without an additional CAR, at doses higher than 90 million cells, exhibited an objective response rate of 72%, with complete responses observed even in patients refractory to prior CD19 CAR-T therapy (Patel et al., 2021). For MM, FT576 integrates a BCMA CAR with CD38 KO, hnCD16, and IL-15 signaling, protecting against MM progression in preclinical studies (Dhakal et al., 2022; Goodridge et al., 2021). Moreover, CNTY-101, an iPSC-derived CAR-NK cell product incorporating a CD19 CAR, secreted IL-15, an epidermal growth factor receptor (EGFR)-truncation safety switch, beta-2 microglobulin (B2M) and class II major histocompatibility complex transactivator (CIITA) KOs, and an HLA-E knockin induced tumor shrinkage, including a durable complete response lasting five months, and tumor microenvironment analyses revealed enhanced adaptive immune activation following the infusion (Patel et al., 2024).

The safety profile of iPSC-derived CAR-engineered therapies remains favorable, with limited severe toxicities reported across clinical trials. FT819 was well tolerated, with no dose-limiting toxicities (DLTs), immune effector cell-associated neurotoxicity syndrome (ICANS), or GvHD, though mild cytokine release syndrome (CRS) was observed in three patients (Mehta et al., 2022a). Similarly, iCAR-NK products FT522, FT516, FT576, and CNTY-101 exhibited minimal toxicity, with no DLTs, ICANS, or GvHD and CRS restricted to grade 1–2 in most cases (Bachanova et al., 2024; Dhakal et al., 2022; Patel et al., 2021, 2024). FT596, evaluated in a larger cohort, reported low-grade CRS in 12% of patients, with no neurotoxicity events (Bachanova et al., 2021; Ghobadi et al., 2025). Transient cytopenias, including neutropenia and thrombocytopenia, were common with FT522 and FT516 but remained manageable (Bachanova et al., 2024). Importantly, CNTY-101 did not elicit functional anti-drug antibodies (ADAs) and was well tolerated across multiple dosing regimens (Patel et al., 2024). These findings support the continued development of iCAR-T and iCAR-NK therapies, with ongoing dose-escalation studies poised to refine optimal therapeutic strategies and expand their clinical applications.

## Challenges in iPSC-derived cell therapy and potential solutions

**Figure 3:**
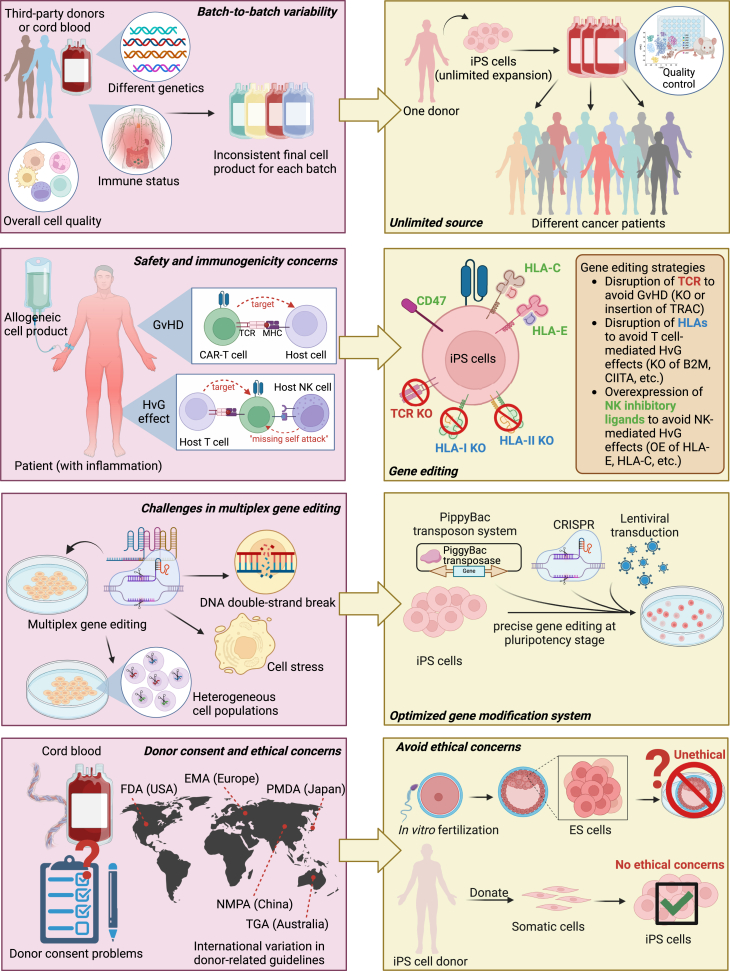
Challenges in allogeneic CAR cell therapy and potential solutions offered by iPSC technology This figure illustrates four key challenges associated with allogeneic CAR cell therapy: batch-to-batch variability, safety and immunogenicity concerns, complexities in multiplex gene editing, and donor-related ethical and consent issues. Corresponding solutions provided by iPSC-derived CAR cells include an unlimited and standardized cell source, enhanced genome editing feasibility, optimized gene modification strategies, and reduced ethical concerns related to donor dependency.

Despite the transformative potential of iPSCs in allogeneic CAR cell therapy, several challenges must be addressed before clinical translation can be fully realized. In the following, we explore these issues and potential solutions (Figure 3).

### Genetic instability

One major concern in iPSC-derived therapies is genetic instability. iPSCs are prone to acquiring chromosomal abnormalities and mutations during reprogramming and prolonged culture, which can compromise their safety and therapeutic efficacy (Madrid et al., 2024). For example, during the reprogramming process, cellular metabolism undergoes a shift from oxidative phosphorylation to aerobic glycolysis, resulting in increased oxidative stress within the cells and consequently elevating the risk of genetic alterations (Hawkins et al., 2016; Steichen et al., 2019; Zhou et al., 2016). These aberrations may lead to functional impairments, loss of differentiation potential, or even oncogenic transformation, posing significant risks for clinical applications (Dashtban et al., 2021; Doss and Sachinidis, 2019). To mitigate these risks, rigorous quality control measures must be implemented, including advanced genomic and transcriptomic screening techniques such as whole-genome sequencing, karyotyping, and single-cell RNA sequencing (Bassett, 2017; Hunt et al., 2023; Kitano et al., 2022). Optimizing reprogramming techniques through non-integrative methods, including episomal vectors, negative-strand RNA Sendai virus, and synthetic mRNA, can reduce insertional mutagenesis and the risk of genomic instability (Madrid et al., 2024). Chemical reprogramming using small molecules like valproic acid (VPA), BRG1 inhibitors, RepSox, and CHIR99021 modulates histone modifications and DNA methylation to reprogram the epigenome to a pluripotent state, providing a non-integrative alternative to traditional reprogramming methods that avoids genomic modification risks (Guan et al., 2022; Liuyang et al., 2023; Wang et al., 2023). Furthermore, the selection of iPSCs from genetically stable donors, termed “super donors,” can enhance reproducibility and safety while limiting inter-individual differences (Al Abbar et al., 2020; Karagiannis et al., 2018; Liu et al., 2024).

### Teratoma and tumorigenic risks

Another pressing issue is the potential for teratoma formation due to the pluripotent nature of iPSCs. If undifferentiated cells persist within a therapeutic product, they may give rise to teratomas, tumors comprising tissues from all three germ layers, which remains a significant safety concern in iPSC-based therapies (Madrid et al., 2024; Yamanaka, 2020). In addition to teratoma, proliferative non-iPSC cells present in terminally differentiated cell populations can pose tumorigenic risks such as malignant transformation, ectopic tissue formation, and uncontrolled proliferation, potentially leading to oncogenesis or the emergence of tumor-initiating cells within the grafted tissue (Lee et al., 2013; Wang, 2023). These risks underscore the importance of thorough purification and quality control in iPSC-derived cell therapies. Ensuring complete differentiation prior to transplantation with strict differentiation protocols is critical for eliminating residual undifferentiated cells (Chang et al., 2014; Jing et al., 2022; Minagawa et al., 2018; Themeli et al., 2013; Yamanaka, 2020). Additionally, cell sorting strategies, such as fluorescence-activated or magnetic-activated cell sorting, can selectively remove undifferentiated iPSCs from the final product (Sun et al., 2024). Another promising approach is the integration of inducible suicide gene systems, such as introducing an inducible caspase-9 suicide gene immediately downstream of *NANOG*: this genetic configuration allows killing only iPSCs to prevent teratoma formation, which can be activated to selectively eliminate residual undifferentiated cells (Ando et al., 2022; Furukawa et al., 2022; Martin et al., 2020). Other methodologies for detecting residual pluripotency include highly sensitive techniques such as droplet digital PCR (ddPCR), which allows for precise quantification of pluripotency-associated transcripts at very low abundance and has already been implemented in clinical trial settings (Kuroda et al., 2015; Yasuda et al., 2024).

### Scalability and standardization

The large-scale manufacturing of iPSC-derived CAR cells also presents significant logistical and technical challenges. Conventional expansion methods often lead to batch variability, limiting the reliability and reproducibility of clinical products (Coll et al., 2018; Matsa et al., 2016; Nicholson et al., 2022; Vatine et al., 2019). Additionally, generating sufficient cell numbers for therapeutic applications requires robust, standardized protocols. One solution to these challenges is the use of bioreactor-based expansion, which enables the automated, large-scale production of iPSC-derived cells with improved yield and consistency (Nogueira et al., 2021). Ensuring compliance with good manufacturing practice regulations is also crucial, as standardized culture conditions and well-defined manufacturing workflows enhance reproducibility and safety (Laurent et al., 2021). Moreover, the development and implementation of xeno-free, recombinant matrix proteins, such as recombinant laminin and fibronectin, have provided a fully defined and contaminant-free platform. These advancements in research or clinical grade significantly reduce batch-to-batch variability (Alidadi et al., 2024; Kasai et al., 2020).

In addition to these challenges, off-target effects during gene editing remain a significant concern in the development of iPSC-derived CAR cell therapies. To mitigate these risks, several strategies have been developed. These include the use of high-fidelity gene-editing tools, such as engineered Cas9 variants (e.g., SpCas9-HF1, eSpCas9, and HypaCas9), which exhibit reduced off-target activity while maintaining on-target efficiency (Chen et al., 2017; Slaymaker et al., 2016; Tsai et al., 2016). Additionally, base editors and prime editors offer precise genome modifications without introducing double-stranded breaks, thereby further minimizing off-target events (Chen and Liu, 2023). Optimization of guide RNA design, such as the use of truncated guide RNAs (tru-gRNAs), also enhances specificity and reduces unintended genomic alterations (Fu et al., 2014).

## Conclusion

Despite the transformative potential of iPSCs in allogeneic CAR cell therapy, several key challenges, including genetic instability, tumorigenicity, scalability, and clinical validation, must be addressed to enable safe and effective clinical translation. To ensure genetic stability, comprehensive genomic surveillance should be implemented throughout the reprogramming, gene-editing, and expansion phases. This includes routine whole-genome sequencing, karyotyping, and copy-number variation analysis to detect and eliminate aberrant clones early. Additionally, the use of non-integrating reprogramming methods, such as Sendai virus, mRNA, or episomal vectors, can minimize the risk of insertional mutagenesis. High-quality iPSC clones with stable karyotypes and epigenomic profiles should be thoroughly screened and banked for downstream applications (Jo et al., 2020; Rohani et al., 2018). To mitigate tumorigenicity, it is essential to establish robust, well-defined differentiation protocols that ensure complete maturation into CAR-expressing effector cells, thereby minimizing the presence of residual undifferentiated iPSCs. Purification strategies incorporating positive selection markers (e.g., CD3 and CAR) and negative selection for pluripotency markers (e.g., SSEA-4 and TRA-1-81) can enhance product safety. Incorporation of fail-safe mechanisms, such as inducible suicide genes (e.g., iCaspase-9 or Herpes Simplex Virus 1-thymidine kinase), provides an added layer of control to eliminate aberrant cells post-infusion if necessary. Addressing scalability and manufacturing requires transitioning from traditional adherent culture systems to scalable, suspension-based bioreactor platforms suitable for large-scale iPSC expansion and differentiation. Closed, automated systems compliant with current good manufacturing practices should be employed to ensure consistency, sterility, and traceability across manufacturing batches. Moreover, differentiation protocols should be optimized to reduce time and complexity through the use of cytokine cocktails, lineage-specifying transcription factors, or three-dimensional culture systems. For clinical validation, standardized release criteria encompassing phenotypic identity, functional potency, viability, and safety must be clearly defined and rigorously tested for each CAR-iPSC-derived product. Every step of the manufacturing process, from reprogramming to the final cell product, should adhere to clinical-grade quality control and documentation standards to facilitate regulatory approval and ensure patient safety (Babu et al., 2023; Madrid et al., 2024).

In addition, notable biological differences exist between somatic and iPSC-derived T and NK cells. Somatic T cells typically exhibit an effector memory or terminal effector phenotype, with their cytotoxicity and proliferative capacity influenced by donor variability and prior antigen exposure. In contrast, iPSC-derived T cells can be generated with a more stem-like phenotype, such as naive or central memory subsets, which may confer enhanced proliferative potential, persistence, and functional responsiveness (Patel et al., 2019). iPSC-derived NK cells commonly display an activated phenotype characterized by high expression of CD16, NKG2D, and natural cytotoxicity receptors (e.g., NKp30, NKp44, and NKp46), contributing to potent antitumor activity (Cichocki et al., 2020). Moreover, while somatic T cells are polyclonal and subject to variability in transgene integration and TCR repertoire, iPSC-derived T and NK cells can be clonally selected and uniformly engineered at the pluripotent stage. This allows for precise genome editing, such as TCR KO, CAR insertion, or HLA modification, and the generation of standardized, homogeneous therapeutic products. Nonetheless, challenges remain in the differentiation of fully functional T cells from iPSCs, particularly in the establishment of memory T cell populations (Netsrithong et al., 2024). Addressing these limitations is critical for the advancement of iPSC-based immunotherapies. Continued innovation in gene editing, manufacturing strategies, and preclinical validation will collectively enhance the safety, efficacy, and clinical applicability of iPSC-derived CAR-engineered cell therapies. iPSC technology may transform adoptive immunotherapy, fostering innovation and expanding the potential of personalized medicine. Beyond CAR-T and CAR-NK therapies, iPSC platforms enable the generation of diverse immune cell types, allowing multiplex gene engineering and the development of next-generation, multi-functional cell products. This versatility opens opportunities for treating not only cancer but also autoimmune and infectious diseases, broadening the clinical impact of iPSC-derived therapies.

## Acknowledgments

Y.-R.L. is supported by a UCLA MIMG M. John Pickett Post-Doctoral Fellow Award, a CIRM-BSCRC Postdoctoral Fellowship, a UCLA Sydney Finegold Postdoctoral Award, a UCLA Chancellor’s Award for Postdoctoral Research, and a UCLA Goodman-Luskin Microbiome Center Collaborative Research Fellowship Award.

## Author contributions

Y.-R.L. and Y.F. conceptualized the article. Y.F. and Y.C. wrote the manuscript, with supervision by Y.-R.L. All authors reviewed and approved the final manuscript.

## Declaration of interests

The authors declare no competing interests.
